# Thermodynamic and Economic Analysis of an Integrated Solar Combined Cycle System

**DOI:** 10.3390/e20050313

**Published:** 2018-04-25

**Authors:** Shucheng Wang, Zhongguang Fu, Sajid Sajid, Tianqing Zhang, Gaoqiang Zhang

**Affiliations:** 1 Key Laboratory of Condition Monitoring and Control for Power Plant Equipment, North China Electric Power University, Beijing 102206, China; 2 School of Energy Power and Mechanical Engineering, North China Electric Power University, Beijing 102206, China; 3 State Key Laboratory of Alternate Electrical Power System with Renewable Energy Sources, North China Electric Power University, Beijing 102206, China

**Keywords:** solar energy, thermodynamic analysis, exergy destruction, combined cycle power plant, economic analysis

## Abstract

Integrating solar thermal energy into the conventional Combined Cycle Power Plant (CCPP) has been proved to be an efficient way to use solar energy and improve the generation efficiency of CCPP. In this paper, the energy, exergy, and economic (3E) methods were applied to the models of the Integrated Solar Combined Cycle System (ISCCS). The performances of the proposed system were not only assessed by energy and exergy efficiency, as well as exergy destruction, but also through varied thermodynamic parameters such as *DNI* and *T*_a_. Besides, to better understand the real potentials for improving the components, exergy destruction was split into endogenous/exogenous and avoidable/unavoidable parts. Results indicate that the combustion chamber of the gas turbine has the largest endogenous and unavoidable exergy destruction values of 202.23 MW and 197.63 MW, and the values of the parabolic trough solar collector are 51.77 MW and 50.01 MW. For the overall power plant, the exogenous and avoidable exergy destruction rates resulted in 17.61% and 17.78%, respectively. In addition, the proposed system can save a fuel cost of 1.86 $/MW·h per year accompanied by reducing CO_2_ emissions of about 88.40 kg/MW·h, further highlighting the great potential of ISCCS.

## 1. Introduction

The production of electricity generated by the consumption of coal, oil, and natural gas is still one of the dominating sources of development in the globe [1]. The rapid development of gas power generation demands huge expenditure of natural gas. More importantly, improvement of efficiency leads to lower energy consumption [2]. In this regard, solar energy is considered to be a promising energy in the near future [3,4,5,6]. Solar energy could also provide 11.3% of global electricity by 2050 according to the International Energy Agency (IEA) [7,8]. Following this progress, some solar thermal power plants have been built in many countries like the United States and China [8].

However, solar thermal power generation is facing some constraints such as huge initial investment (heat storage system accounts for 25% of total investment) and low thermal performance [9,10,11]. While integrating the parabolic trough solar field into a conventional Combined Cycle Power Plant (CCPP) leads to significant reductions in the capital and operation and maintenance costs due to utilization of common equipment such as the steam turbine and heat sink.

Initially, Luz Solar International proposed an integrated solar combined cycle system (ISCCS) to increase power plant efficiency and reduce fossil fuel consumption [12,13]. In the system, steam produced by the solar collector plant is not directly used for power generation but to replace the steam in the Heat Recovery Steam Generator (HRSG), and then to continue to do work in the turbines. The ISCCS can avoid the instability of the pure solar thermal power plant which uses solar energy directly. Besides, the ISCCS can save a part of the fuel consumption and improve the efficiency compared with conventional CCPP, since solar energy is free and abundant in nature. Therefore, many ISCCSs have been built in the world including the 75 MW Solar Energy Center in Florida, the 20 MW ISCCS Hassi R’me in Algeria, the 20 MW ISCCS Kuraymat in Egypt, and the 20 MW ISCCS Ain Beni Mathar in Morocco [14,15,16].

Many researches on ISCCS have been done on the basic theory and application to optimize performance. Kelly et al. [17] studied two integrated generations and concluded that producing high-pressure steam for addition to the HRSG is the most efficient way to use solar thermal energy. Li et al. [4] proposed and investigated a two-stage ISCCS with direct steam generation (DSG) technology, and the net solar-to-electricity efficiency and exergy efficiency of the overall system were boosted by 1.2% and 2.5% through this technique compared with the one-stage ISCCS. Zhu [18] utilized a model of an ISCCS to explore the system behavior under different input parameters (ambient temperature and solar thermal input). The modulated approach described that solar hybridization into the CCPP was effective in achieving higher efficiency than that of the steam cycle. In another report, Liu et al. [19] figured out the thermodynamic performance of two solar-biomass hybrid CCPPs under off-design conditions. The annual overall system net solar-to-electric efficiency and energy efficiency resulted in 18.49% and 29.36%, respectively. Additionally, to improve the performance of the gas turbine with high *DNI* and ambient temperature, Montes et al. [20] documented the annual operation of an ISCCS. The better ISCCS results were demonstrated in Las Vegas and Almeria, especially when the solar hybridization was coupled to the CCPP. In addition, Baghernejad et al. [21] used a thermo-economic concept for optimization of an ISCCS. The results show that the objective function (investment cost of equipment) for the optimum operation was reduced by 11% and the electricity cost was lower than the base case. Brodrick et al. [22] revealed that a marked increase in the operating flexibility of the ISCCS is observed when the outlet temperature of the solar heat transfer fluid is allowed to vary over the course of the day. Mabrouk et al. [23] evaluated the performance of ISCCS by thermodynamic analysis, and additionally investigated the performance of the main parameters on solar integration. It revealed that the thermal-to-electrical efficiency drops as the integrated solar rate increases. However, the efficiency can be improved by increasing the mass flow rate of the solar field.

Exergetic analysis has become a key tool and an integral part of thermodynamic assessment in analyzing power generation systems. Fahad et al. [24] analyzed selected thermal systems driven by PTSC. This revealed that the main source of exergy destruction was the solar collector where more than 50% of inlet exergy was destroyed. To the best of our knowledge there are very limited articles based on the energy, exergy analysis, and economic performance assessment of the ISCCS. Zare et al. [3] assessed a combined cycle, which consisted of two organic Rankine cycles and a closed Brayton cycle. The results indicated that an exergy efficiency of more than 30% was achieved. In addition, the system showed a better performance than the others under similar operation conditions. Sorgulu et al. [25] evaluated an ISCCS via thermodynamic analysis and results showed that 151.72 MW output power is generated by recovering exhausted gases and using solar collectors. Rovira et al. [26] revealed that the only-evaporative DSG configuration had a better performance in ISCCS configurations, since it benefitted from both high thermal efficiency in the solar field and low irreversibility in the HRSG.

In this paper, the overall design of ISCCS was analyzed via energy and exergetic methods. Different from previous studies, our work considers the effect of ambient temperature and solar radiation intensity. Our goal is to further evaluate quantitatively the causes and locations of the thermodynamic imperfection in the system, and thus indicate the possibilities of thermodynamic improvement through exergy destruction in each components of ISCCS. Moreover, economic analysis was used to evaluate the economic rationality of the system. Our results provide significant ways to improve energy-saving in ISCCS accompanied by reduced CO_2_ emissions of 88.40 kg/MW·h.

## 2. System Description and Assumptions

### 2.1. System Description

**Figure 1:**
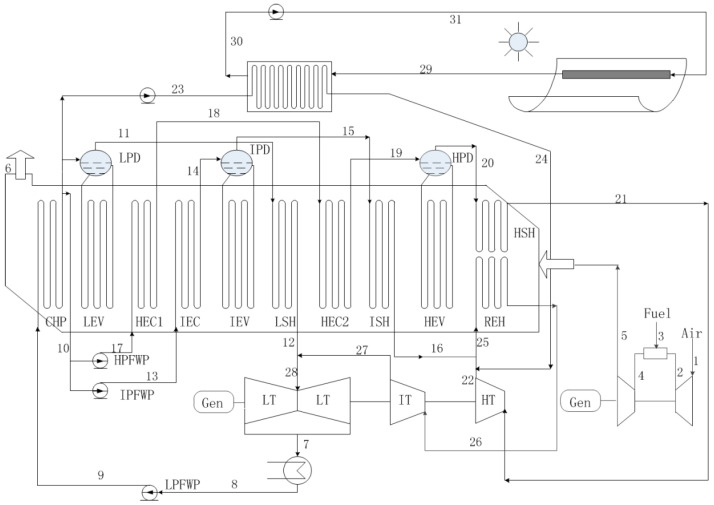
The flowchart of Integrated Solar Combined Cycle System (ISCCS).

The proposed ISCCS consists of a traditional SGT5-4000F including a Siemens V94.3A gas turbine (Zhengzhou, China), a three-pressure HRSG with reheat and a parabolic trough solar collector (PTSC) as depicted in Figure 1.

In the ISCCS, the processes start from the burning of compressed air and fuel in the combustion chamber (CC). The produced gas accompanied by high temperature and pressure further expands in the turbine to deliver useful work. In addition, the flue gas of the gas turbine enters the HRSG to heat feed water to steam. This steam goes to the steam turbine through economizers, evaporators, and super heaters. On the other hand, when the CCPP is integrated with solar energy, a certain amount of feed water from the HRSG will be heated up by solar energy as well.

**Figure 2:**
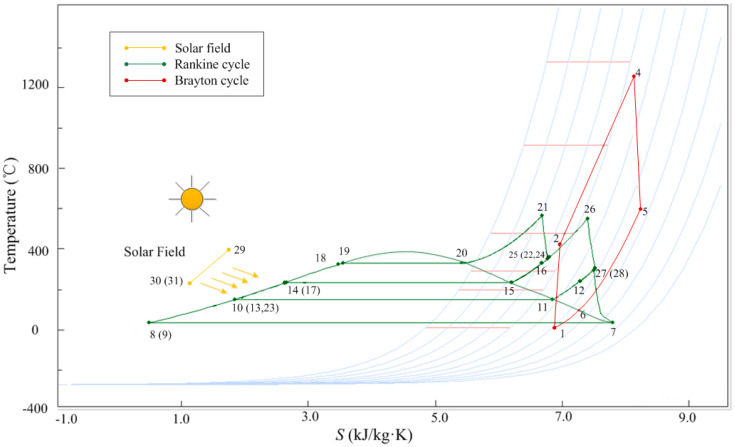
The T-S diagram of ISCCS.

In particular, the solar injection point is a very significant parameter because efficient solar injection leads to a higher solar-to-electric efficiency (*η*_sol-elec_). Additionally, considering the lower temperature difference between the steams before mixing could be effective to overcome the energy losses. Therefore, the superheated steam (358 °C) is generated by the solar collector mixed with the exhaust steam (349 °C) of the high pressure turbine and the intermediate pressure superheated steam (329 °C). Furthermore, the reheater heats up these steams before they are injected back to the intermediate pressure turbine. The T-S diagram of the ISCCS is shown in Figure 2.

Herein, solar energy was used as an auxiliary resource and the superheated steam temperature of the collectors was kept constant. However, the mass flow rate can be changed with the solar radiation intensity. A certain amount of feed water gets heat in the PTSC during sunny periods, therefore, the consumption of natural gas is reduced. However, the integrated power plant operates as a conventional combined cycle (CCPP) during cloudy periods or at night.

### 2.2. Assumptions

The analysis was investigated under assumed various operating conditions as follows:

▪ The temperature was constant for the exhaust gas from the gas turbine.
▪ Air and flue gas were considered as ideal gases, there are no pressure drops within the components.
▪ The fuel of CCPP was natural gas at a lower heating value (*LHV* = 49,015 kJ/kg).
▪ The ambient operating temperature and pressure of the reference environment were 20 °C and 1.0 bar, respectively.

## 3. Mathematical Modeling

Mathematical modeling of the proposed systems is presented in this section. Moreover, the thermodynamics analysis is divided into energy analysis, conventional exergetic analysis, and advanced exergetic analysis.

The incident solar power on the collector system is given by the equation:

(1) $$Q_{s}=N\times A\times DNI$$

where N is the number of collectors and A is the area of collectors.

The energy absorbed by the absorber tube is expressed as:

(2) $$Q_{a}=\eta_{\mathrm{opt}}\times Q_{s}$$

In Equation (2) the optical efficiency of collectors ($\eta_{\mathrm{opt}}$) is further defined by

(3) $$\eta_{\mathrm{opt}}=\eta_{\rho }\times \eta_{\tau }\times \eta_{\alpha }\times \eta_{\gamma }\times \eta_{\phi }\times \eta_{\mu }\times K$$

where $\eta_{\rho }$, $\eta_{\tau }$, $\eta_{\alpha }$, $\eta_{\gamma }$, $\eta_{\phi }$, $\eta_{\mu }$ and *K* are the surface reflectivity of the compound parabolic concentrator, receiver transmissivity, receiver absorption rate, acquisition factor, mirror utilization rate, radiation and convective heat loss efficiency, and correction factor of incident angle, respectively. In addition, we applied the energy efficiency of ISCCS as the ratio of net power output to the total input energy in the power plant.

(4) $$\eta_{\mathrm{ISCCS}}=\frac{W_{\mathrm{net}}}{m_{f}\times LHV+Q_{s}}$$

where $m_{f}$ and *LHV* are the mass flow rate and the lower heat value of the fuel.

The net solar-to-electricity efficiency is defined to evaluate the performance of the solar heat conversion in ISCCS.

(5) $$\eta_{\mathrm{sol}-\mathrm{elec}}=\frac{W_{\mathrm{net}}-W_{\mathrm{ref}}}{Q_{a}}$$

where $W_{\mathrm{ref}}$ is the net power output by the reference system (CCPP) with the same natural gas input.

For the proposed system, the solar heat fraction is used to evaluate the amount of thermal energy provided by the solar field.

(6) $$\chi_{\mathrm{solar}}=\frac{Q_{a}}{m_{f}\times LHV+Q_{a}}$$

The fuel saving fraction for the proposed system is given by the following equation [27]:

(7) $$\chi_{\mathrm{saving}}=\frac{Q_{\mathrm{ref}-\mathrm{fossil}}}{Q_{\mathrm{fossil}}}=\frac{Q_{\mathrm{ref}}-Q_{\mathrm{fossil}}}{Q_{\mathrm{fossil}}}$$

The definition of exergy is a measure of the maximum capacity of a system to perform useful work. Herein, we express exergetic analysis through four distinct parts: kinetic, potential, physical, and chemical exergy. When potential and kinetic exergy are neglected, the exergy balance is expressed as follow [28]:

(8) $$\dot{E}x=\dot{E}x_{\mathrm{ph}}+\dot{E}x_{\mathrm{ch}}$$

where physical and chemical exergy are defined as:

(9) $$\dot{E}x_{\mathrm{ph}}=\dot{m}[(h-h_{0}-T_{0}(s-s_{0})]$$

(10) $$\dot{E}x_{\mathrm{ch}}=\dot{m}[\sum_{i=1}^{n}x_{i}ex_{i}+RT_{0}\sum_{i=1}^{n}x_{i}\ln x_{i}]$$

The complications of the chemical exergy calculation of fuel have been noticed with the above equation. Therefore, the following equation is used for the derivation.

(11) $$\dot{E}x_{f}=\xi \times LHV$$

where LHV is the lower heating value of nature gas and ξ is the ratio of fuel chemical exergy to lower heating value, which can be calculated by the flowing equation:

(12) ξ=1.033+0.0169(y/x)−(0.0698/x)

Furthermore, the projected exergy and absorbed exergy via the collectors were expressed as

(13) $$\dot{E}x_{i}=Q_{i}(1-T_{a}/T_{s})$$

(14) $$\dot{E}x_{c}=Q_{s}(1-T_{a}/T_{r})$$

where $T_{a}$, $T_{s}$, and $T_{r}$ are ambient temperature, solar surface temperature, and the collectors surface temperature, respectively.

Based on the measured spectrum of radiation, the exergy of the solar radiation arriving at the earth was discussed by Petela [29] and Szargut [30]:

(15) $$b_{\omega }=\frac{b}{\pi }\int \int_{\omega }\cos\vartheta \sin\vartheta d\vartheta d\phi$$

where *ω* is the angle at which the sun is visible from the earth, *ϑ* and *φ* are the azimuth and declension angle coordinates, respectively; *b* is the exergy radiation emitted by the sun.

The advanced exergetic analysis was applied on ISCCS. We will analyze the destruction in each component under non-ideal working conditions. Exergy balance of *k*-th is defined as:

(16) $$\dot{E}x_{D,k}=\dot{E}x_{F,k}-\dot{E}x_{p,k}$$

where $\dot{E}x_{D,k}$ is the exergy destruction caused by the irreversibility of components, $\dot{E}x_{F,k}$ and $\dot{E}x_{p,k}$ are the “Fuel” exergy consumed and the “Product” exergy in the process of energy conversion [31,32].

Additionally, for the *k-*th component, the exergy efficiency and destruction rate are defined:

(17) $$\eta_{e}=\dot{E}x_{p,k}/\dot{E}x_{F,k}$$

(18) $$y_{D,k}={\dot{E}}_{D,k}/{\dot{E}}_{F,k}$$

The exergy balance equation for the overall system can be written as:

(19) $${\dot{E}}_{F,\mathrm{tot}}={\dot{E}}_{P,\mathrm{tot}}+\sum_{k}{\dot{E}}_{D,k}+{\dot{E}}_{L,\mathrm{tot}}$$

where ${\dot{E}}_{F,\mathrm{tot}}$, ${\dot{E}}_{P,\mathrm{tot}}$, ${\dot{E}}_{L,\mathrm{tot}}$ are the total “fuel” exergy input in the system, total “product” exergy, and the exergy lost for the system.

Moreover, various components interact with each other in a complex system, therefore, the exergy destruction is split into endogenous (${\dot{E}}_{D,k}^{\mathrm{EN}}$) and exogenous (${\dot{E}}_{D,k}^{\mathrm{EX}}$) [14,33]. In order to estimate the endogenous exergy destruction of the *k*-th component, the *k*-th component was defined operating under real conditions, while other components of the proposed system operate under theoretical conditions (as shown in Table 1) [34], the result is endogenous of the *k*-th component. Then, the exogenous exergy destruction can be estimated by the following equation:

(20) $$\dot{E}x_{D,k}^{\mathrm{EX}}=\dot{E}x_{D,k}-\dot{E}x_{D,k}^{\mathrm{EN}}$$

Besides, the part of exergy destruction which cannot be reduced is called unavoidable exergy destruction ($\dot{E}x_{D,k}^{\mathrm{UN}}$), and the other part that can be reduced is avoidable exergy destruction ($\dot{E}x_{D,k}^{\mathrm{AV}}$) [34]. Some assumptions (as shown in Table 1) based on Petrakopoulou et al. [31] were used to calculate the unavoidable exergy destruction of the *k-*th component, which was defined by the experience and knowledge of the author on CCPP. Then, the avoidable exergy destruction of the *k-*th component can be estimated by [34]:

(21) $$\dot{E}x_{D,k}^{\mathrm{AV}}=\dot{E}x_{D,k}-\dot{E}x_{D,k}^{\mathrm{UN}}$$

The output results of the above approaches provide a thorough understanding of the system energy-saving, improving components performances, and reducing irreversibility losses in the working process.

## 4. Results and Discussion

### 4.1. Model Validation

The models of CCPP and PTSC were built on Ebsilon^®^ Professional (12.05, STEAG company, Essen, Germany), which is widely used in power plant design, evaluation, optimization, and other thermal cycle processes. In order to validate the accuracy of the simulation process of the proposed model, a series of main parameters were selected. The main thermodynamic parameters of design values based on the SGT5-4000F running data and simulation values of CCPP are shown in Table 2. Herein, we noticed that deviations between models and the designed system were of an acceptable scope, highlighting the potential of the proposed models for further optimization.

A LS-2 trough solar collector with single axis tracking and uniformed on a north-south line was chosen to track the sun radiation from east to west as the case study. The main design parameters of PTSC are listed in Table 3. Our works were carried out (ambient temperature of 20 °C, wind speed of 2.2 m/s) on 21st of June in Zhengzhou (34.7° N, 113.7° E). Herein, the total incident radiation on the collectors was about 183 MW with total energy absorption of 99.72 MW. The mass flow rate of oil in the collectors was 229.57 kg/s. The temperature of the feed water and superheated steam from the solar collector were 149.5 °C and 358.5 °C, respectively.

### 4.2. Energy and Conventional Exergetic Analysis

**Figure 3:**
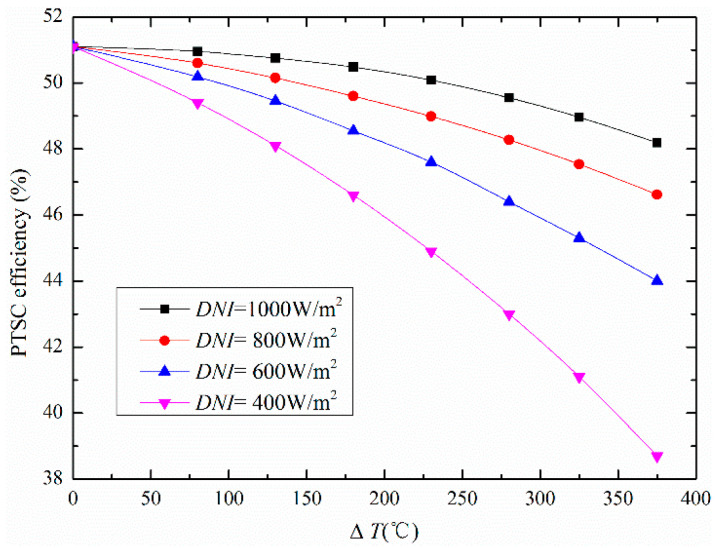
Parabolic trough solar collector (PTSC) efficiency variation under different *DNI* and Δ*T* values.

The energy, exergetic and economic (3E) analysis of the proposed ISCCS were investigated via thermodynamic variables for selected material streams as listed in Table 4. In the energy analysis we showed that the heat efficiency of parabolic collectors is related to the *DNI* and the difference value between operating temperature and ambient temperature (Δ*T* = *T* − *T*_a_). The dependence of PTSC efficiency with the *DNI* and Δ*T* is displayed in Figure 3. It can be seen that the PTSC efficiency drops at high operating temperature (*T*), however a higher *DNI* value results in an efficient performance. Herein, the curves in Figure 3 were obtained under the normal incident angle of solar light condition. It was observed that the maximum heat collection efficiency occurred when *T* is equal to the *T*_a_, which means the thermal efficiency and optical efficiency are similar.

**Figure 4:**
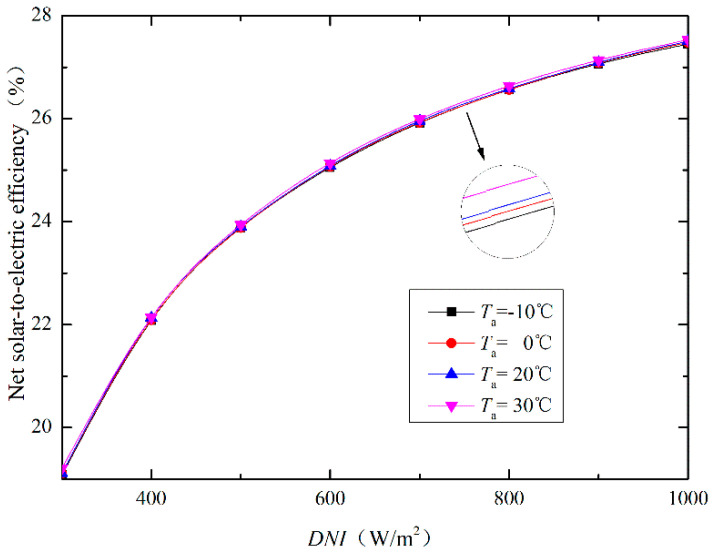
Net solar-to-electric efficiency curves with varying *DNI* and *T*_a_ values.

Additionally, the net solar-to-electric efficiency ($\eta_{\mathrm{sol}-\mathrm{elec}}$) shows efficient performance with higher value of *DNI* under each *T*_a_ as plotted in Figure 4. There is a rapid response of the $\eta_{\mathrm{sol}-\mathrm{elec}}$ when the *DNI* is less than 500 W/m^2^, and a slow response when *DNI* is greater than 500 W/m^2^. In contrast, the *T*_a_ shows little influence on the $\eta_{\mathrm{sol}-\mathrm{elec}}$. Furthermore, the effect of *DNI* on $\eta_{\mathrm{sol}-\mathrm{elec}}$ is greater than that of *T*_a_. The higher value of *DNI* increases the mass flow rate of PTSC, and thus provides more solar energy to the system.

**Figure 5:**
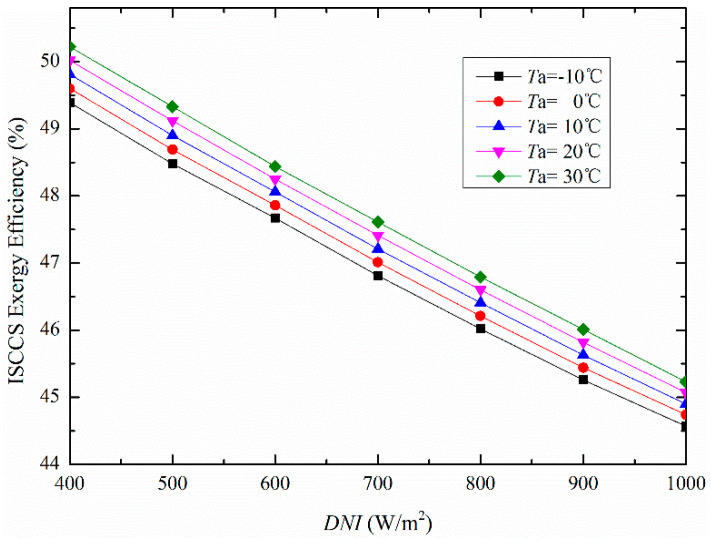
Illustration of ISCCS exergy efficiency under varying *DNI* and *T*_a_ values.

The influence of *DNI* on overall plant exergy efficiency under different *T*_a_ is plotted in Figure 5. The simulation results revealed that the ISCCS exergy efficiency consistently increases with *T*_a_, however, it drops with the increase of *DNI*. It is important to notice that the solar energy density utilized through PTSC is lower than the fossil fuel consumed in CCPP. Although, the efficiency of PTSC is lower than that of CCPP, however as a natural source of free energy it is desirable for the community of ISCCS.

**Figure 6:**
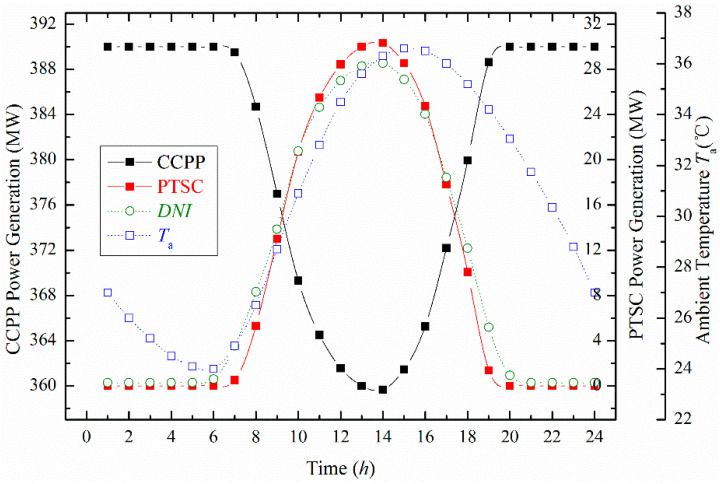
Different power generation produced by Combined Cycle Power Plant (CCPP) and PTSC varying with time.

We further investigated that a part of natural gas consumption could be reduced with the increase of solar energy generation in the proposed ISCCS. Figure 6 shows that the power generation contributed from PTSC increases with the *DNI*. The highest PTSC power generation from 0 MW to 30 MW was obtained with an increased *DNI* value (864 W/m^2^). The corresponding power generation contributed from solar energy was about 7.7% compared to the overall plant power output. At the same time, the CCPP power generation drops from 390 MW to 360 MW when the total power generation of the ISCCS remains constant.

**Figure 7:**
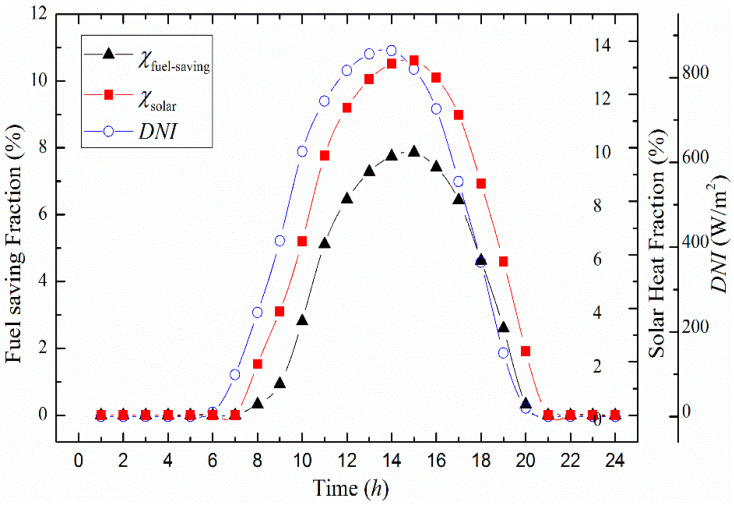
Fuel-saving fraction and solar heat fraction varying with time.

The overall fuel-saving fraction and solar heat fraction values for transient behavior of solar irradiance on 21st June are illustrated in Figure 7. It has been noted that the higher the performance of *DNI*, the higher are the fuel-saving fraction and solar heat fraction. Also, the highest values of 7.86% and 13.26% are achieved at solar noon times, respectively.

**Figure 8:**
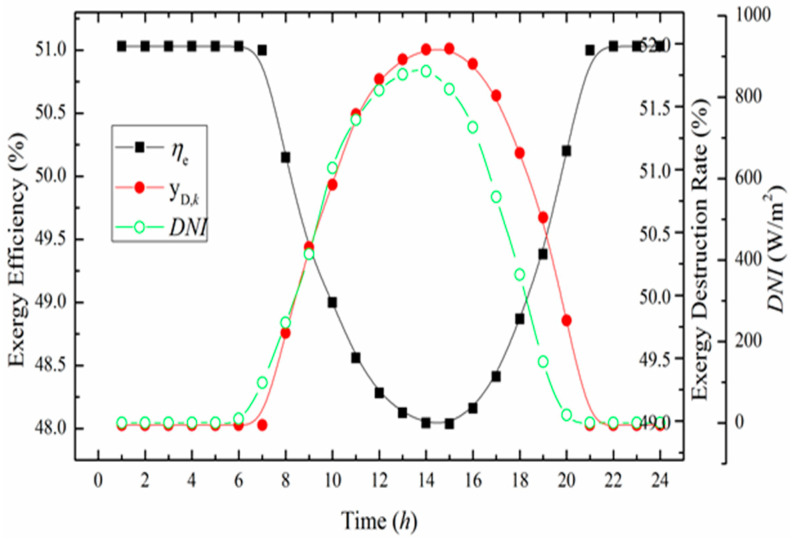
Fuel-saving fraction and solar heat fraction varying with time.

The exergy efficiency and exergy destruction rate of the overall system varying with time are shown in Figure 8. It is observed that the higher the *DNI* the lower the exergy efficiency. Besides, the tendency of the exergy destruction rate shows the opposite performance. For the reason that the energy density of solar energy is lower than the fossil fuel, the more the solar energy input of the ISCCS, the lower the exergy efficiency. However, as a natural free source, integrating solar energy into a conventional CCPP can save fuel cost.

Another important analysis of the ISCCS was carried out via exergetic analysis. It is known that energy analysis is based on the first law of thermodynamics, while exergetic analysis is based on both the first and second law of thermodynamics. Besides, exergetic analysis is effective in evaluating quantitatively origins and sites of thermodynamic deficiencies in the energy system, thus revealing the possibilities of thermodynamic enhancement. Moreover, the conclusions from the exergetic analysis have a vital role in the existing processes improvement. The calculated exergy variables for selected main components are shown in Table 5.

**Figure 9:**
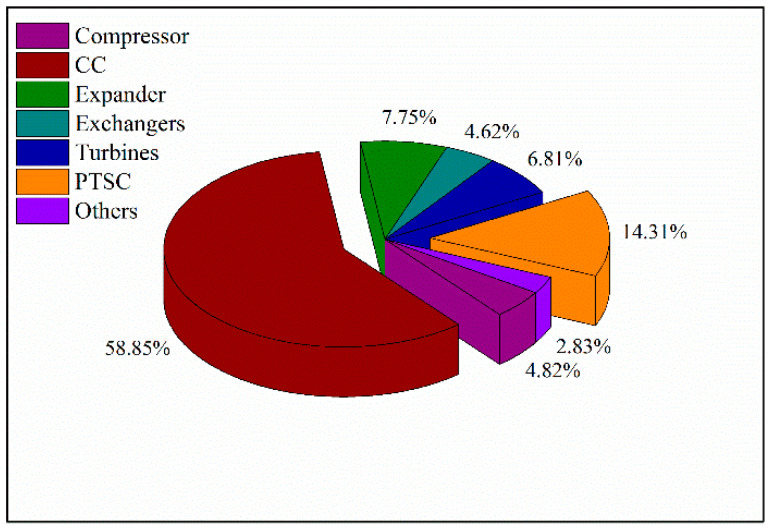
Pie chart of exergy destruction with different components of the ISCCS.

Furthermore, to make meaningful our proposed system, the exergy destruction within different parts of the ISCCS was analyzed as shown in Figure 9. The CC resulted in the largest exergy destruction of 58.85%. This largest exergy destruction was not only caused by the low fuel temperature before burning but also the process of fuel heating to get to the fire point. The energy loss in the oil-water heat exchanger and the heat transfer process within the long pipe-lines region of the PTSC caused the second largest exergy destruction of 14.31%. Therefore, it is clear from the above analyses that the CC and PTSC should be addressed for further energy conservation.

### 4.3. Advanced Exergetic Analysis

**Figure 10:**
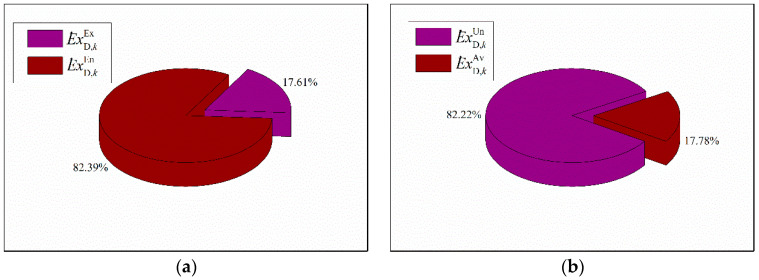
The exergy destruction of ISCCS. (**a**) Endogenous and exogenous exergy destruction. (**b**) Avoidable and unavoidable exergy destruction.

The advanced exergetic method on ISCCS was further implemented to better understand the causes of exergy destruction thoroughly in every component. Herein, the exergy destruction was split into exogenous/endogenous and unavoidable/avoidable parts. While the endogenous exergy destruction is independent of external factors, it can be caused by the irreversible losses. On the other hand, exogenous exergy destruction is relevant to the operation condition and the interaction effects among components. The diagrams of overall exogenous/endogenous and unavoidable/avoidable exergy destruction of ISCCS are presented in Figure 10. It can be observed that the endogenous exergy destruction rate resulted in 82.39% while the exogenous exergy destruction was 17.61%. This approach also revealed that most irreversible exergy destruction was caused by the component itself and was independent of external factors, which reveals that the system topology contributes largely to its exergy destruction. Besides, we can also observe that unavoidable exergy destruction resulted in 82.22% while the avoidable exergy destruction was 17.78%. It should be noted that the unavoidable exergy destruction indicates that it cannot be reduced under the current technological or economical constraints. However, the proportions of unavoidable and exogenous parts differ significantly with different types of components.

**Figure 11:**
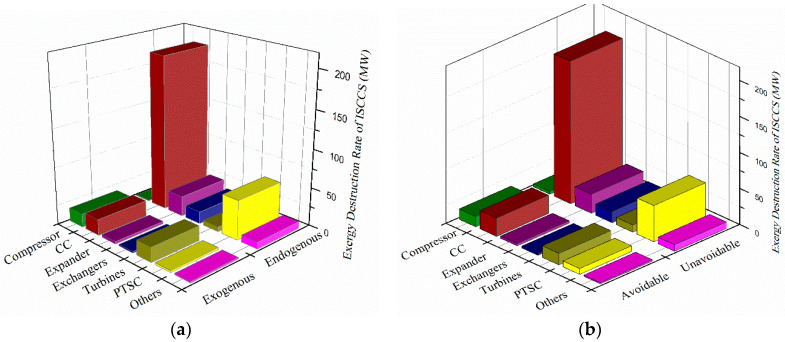
The exergy destruction rates of main components. (**a**) Endogenous and exogenous exergy destruction rate. (**b**) Avoidable and unavoidable exergy destruction rate.

**Figure 12:**
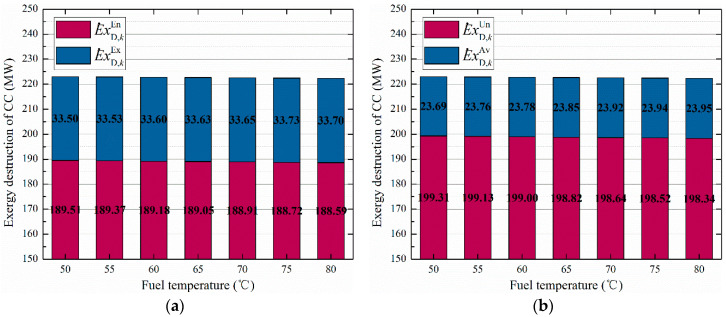
The exergy destruction of CC varies with fuel temperature. (**a**) Endogenous and exogenous exergy destruction; (**b**) Avoidable and unavoidable exergy destruction.

The exergy destruction rates of main components are shown in Figure 11. It can be seen that the endogenous and unavoidable exergy destruction in CC (199.31 MW and 189.51 MW) are the highest, followed by the PTSC (51.77 MW and 50.01 MW). However the CC also has a large avoidable exergy destruction of 33.50 MW, and thus it can be reduced by improving the operating conditions, such as preheating the fuel before burning in CC. Besides, the largest exogenous exergy destruction rates occurred in the turbines (74.83%) and compressor (95.24%), which can be improved by reducing both their inherent irreversibility and the inefficiency. Additionally, we presented the exergy destruction (unavoidable/avoidable and exogenous/endogenous) of CC varying with the fuel temperature, as shown in Figure 12. It can be observed that with the fuel temperature increase from 55 °C to 80 °C, the total exergy destruction of CC decreased 0.72 MW, and the endogenous and unavoidable exergy destruction decreased 0.91 MW and 0.68 MW, respectively.

The obtained information by spitting the exergy destruction into endogenous/exogenous and avoidable/unavoidable parts help us better understand the potential for improving and the interdependencies in the components. In addition, all the exogenous, endogenous, avoidable, and unavoidable exergy destruction values are positive which means that the performance of components improves with the performance enhancement of the remaining system components.

### 4.4. Economic Analysis

The thermodynamic performance of the overall design and the components in the proposed system has been discussed above, besides, a preliminary economic analysis needs to be assessed as well. Herein, considering the cost of PTSC, operation and maintenance, the levelized energy cost of the electricity (*LCOE*) was defined and used for the basic economic evaluation criteria, which can be formulated as [9,10]:

(22) $$LCOE=\frac{LC_{\mathrm{LNV}}+LC_{O\&M}}{E_{\mathrm{annual}}}$$

where $LC_{O\&M}$ is the operation and maintenance costs and $LC_{\mathrm{LNV}}$ is the levelized costs of the investment, which can be calculated as [9,10]:

(23) $$LC_{\mathrm{LNV}}=CRF\times INV$$

(24) $$CRF=\frac{i_{\mathrm{eff}}\times {\left(1+i_{\mathrm{eff}}\right)}^{n}}{{\left(1+i_{\mathrm{eff}}\right)}^{n}-1}$$

where *INV* is the total equipment investment and *CRF* is the capital recovery factor and $i_{\mathrm{eff}}$ is the effective discount and *n* is the economic life of the system.

The annual cost of the proposed system can be calculated by the formula:

(25) $$A=LC_{O\&M}\times b\times \left(b^{N}-1/b-1\right)$$

(26) b=(1+e)/(1+r)

where *A* is the annual cost, *N* is the time in year, *e* is the inflation rate figure, *r* is the effective discount rate.

The economic evaluations show that the specific investment of the solar field is about 286.57 $/m^2^, the operation and maintenance cost for new equipment is 2% of the total investment. In addition, the effective discount rate is 7% and economic life is about 30 years as the economic analysis results show in Table 6. Furthermore, the reduction of fuel cost is 1.86 $/MW·h due to the solar energy input to the system. On the other hand, CO_2_ emissions can be reduced by about 88.40 kg/MW·h. Additionally, from such an analysis it can be concluded that the ISCCS is not only desirable for economic benefits, but also for reducing global warming than conventional CCPP, and thus, it provides better commercial competitiveness.

**Figure 13:**
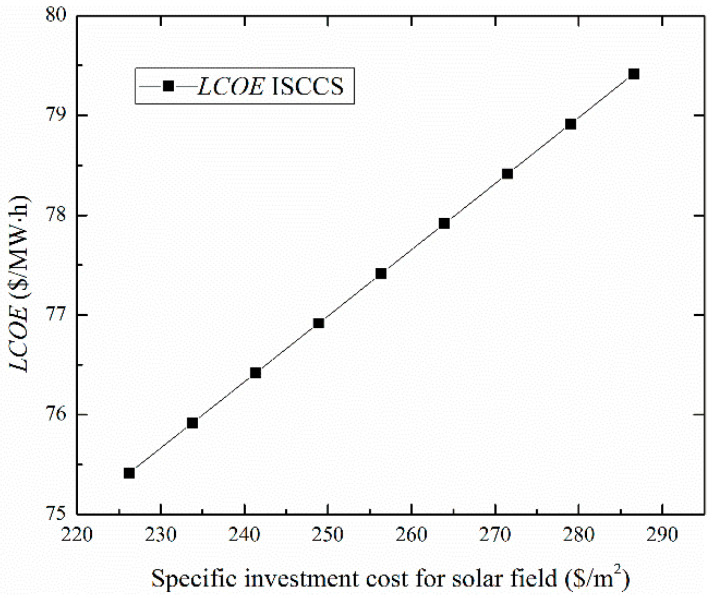
The levelized energy cost of the electricity (LCOE) values for ISCCS as a function of the specific cost on the solar field.

**Figure 14:**
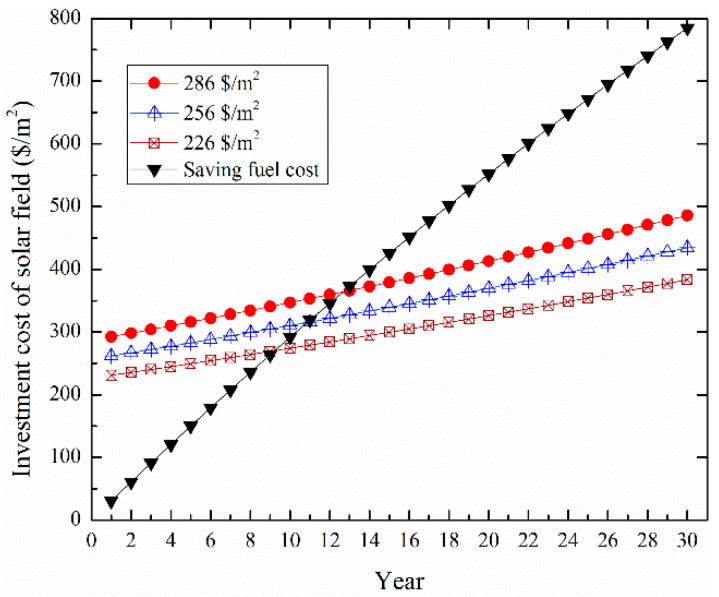
The predicted cumulative system cost of the solar field.

Figure 13 shows the variation of the LCOE values for the ISCCS as a function of the specific investment cost of the solar field. It was found that the LCOE still falls as the decrease of solar field specific investment cost falls. Figure 14 shows the predicted cumulative system cost for the solar field as a function of time in years for three different solar field area prices of 226, 256 and 286 $/m^2^. The break points (payback time) range from 9 to 13 years. The current cost of the solar field in general is expected to decrease dramatically with mass production and this may then make such a system more economically viable. Furthermore, the proposed system provides a cost effective way against the high price of natural gas. Indeed, the advantages of an ISCCS over a CCPP are clearer when CO_2_ emissions are considered and the ISCCS would be more economical than a CCPP when considering the carbon price.

## 5. Conclusions

In this study, the investigations through energy, exergetic, and economic (3E) methods were aimed on the ISCCS, which includes conventional CCPP and PTSC. The varied thermodynamic properties (including power generations, fuel saving fraction, solar heat fraction, energy efficiency and exergy destruction rate) of the proposed system were discussed. Additionally, the exergy destruction was split into exogenous/endogenous and unavoidable/avoidable parts to better understand the real potential of the overall system and components. Besides, an economic analysis was carried out to estimate the cost-effectiveness. From the obtained results the following can be concluded about this study:

▪ The efficiency of PTSC was influenced by the *DNI* and ∆*T* (*T* − *T*_a_), in such way that the effect of *DNI* on the net solar-to-electric efficiency was greater than that of *T*_a_. Thus, the highest PTSC power generation from 0 MW to 30 MW was obtained with increased *DNI* values (864 W/m^2^).
▪ For the overall power plant, the exogenous and endogenous exergy destruction resulted in 17.61% and 82.39%, while the unavoidable and avoidable were 82.22% and 17.78%, respectively. The largest endogenous and unavoidable exergy destruction were displayed in CC, followed by PTSC. Besides, the turbines and compressor have the largest exogenous exergy destruction rates of 74.83% and 95.24%, respectively.
▪ A reduced cost of fuel consumption of about 1.86 $/MW·h and minimized CO_2_ emissions of 88.40 kg/MW·h were achieved by the proposed system, which further highlighted the great potential of ISCCS. In particular, the ISCCS is desirable for both fuel-saving and global warming control at low cost.

## Figures and Tables

**Table 1:** The basic parameters for advanced exergetic analysis.

Component, *k*	Real Condition	Theoretical Condition	Unavoidable Condition
Compressor	$\eta_{\mathrm{th}}=98\%$	$\eta_{\mathrm{th}}=100\%$	$\eta_{\mathrm{th}}=99\%$
CC	$Q_{L}=2\%$	$Q_{L}=0\%$	$Q_{L}=0\%$
Expander	$\eta_{\mathrm{th}}=98\%$	$\eta_{\mathrm{th}}=100\%$	$\eta_{\mathrm{th}}=99\%$
Turbines	$\eta_{\mathrm{th}}=95\%$	$\eta_{\mathrm{th}}=100\%$	$\eta_{\mathrm{th}}=99\%$
	$\eta_{\mathrm{is}}=88\%$	$\eta_{\mathrm{is}}=100\%$	$\eta_{\mathrm{is}}=97\%$
Pumps	$\eta_{\mathrm{th}}=95\%$	$\eta_{\mathrm{th}}=100\%$	$\eta_{\mathrm{th}}=99\%$
	$\eta_{\mathrm{is}}=80\%$	$\eta_{\mathrm{is}}=100\%$	$\eta_{\mathrm{is}}=97\%$

**Table 2:** Main parameters of SGT5-4000F.

Parameters	Siemens	Simulation	Units
Capacity	390	390	MW
Main steam	12.5/566/72.6	12.6/567/73.8	MPa/°C/kg·s^−1^
Reheated steam	2.99/551/85.6	2.91/551/86.7	MPa/°C/kg·s^−1^
Low-pressure steam	0.45/239/12.3	0.46/239.9/12.9	MPa/°C/kg·s^−1^
Gas turbine exhaust	590/643	590.6/646	°C/kg·s^−1^
Ambient temperature	20	20	°C
Exhaust gas temperature	90	90.9	°C

**Table 3:** Main parameters of the parabolic trough solar collectors.

Parameters	Values	Units
Length	150	m
Width	5.76	m
Temperature of water in/out of receiver	149.5/358.5	°C
Number of collectors	280	-
Surface reflectivity $\eta_{\rho }$	0.92	-
Receiver transmissivity $\eta_{\tau }$	0.90	-
Receiver absorption $\eta_{\alpha }$	0.91	-
Acquisition factor $\eta_{\gamma }$	0.93	-
Mirror utilization $\eta_{\Phi }$	0.91	-
Radiation and convective heat loss efficiency $\eta_{\mu }$	0.90	-

**Table 4:** Calculated thermodynamic variables for selected material streams.

Stream, *j*	$T_{j}$ (°C)	$P_{j}$ (bar)	$m_{j}$ (kg/s)	$e_{j}$ (kJ/kg)	$h_{j}$ (kJ/kg)	$E_{j}$ (MW)
1	20.00	1.00	631.89	0.00	20.29	0.000
2	420.24	17.00	631.89	396.45	437.14	250.513
3	50.00	17.00	13.80	51848.42	109.05	715.249
4	1225.46	17.00	645.68	1150.34	1445.68	742.754
5	590.43	1.04	645.68	320.73	653.65	207.088
6	90.92	1.01	645.68	22.62	95.91	14.607
7	32.88	0.05	124.17	248.14	2377.14	30.810
8	32.88	0.05	124.17	7.58	137.77	0.941
9	32.92	4.70	124.17	8.06	138.35	1.000
10	118.99	4.65	124.17	85.31	499.70	10.593
11	149.12	4.65	13.72	875.06	2744.86	12.003
12	239.84	4.60	13.72	954.91	2941.46	13.098
13	149.56	29.90	82.72	130.66	631.90	10.809
14	230.58	29.85	82.72	278.53	992.97	23.040
15	233.58	29.85	16.22	1112.97	2803.26	18.055
16	329.47	29.80	16.22	1245.98	3067.68	20.212
17	233.28	126.10	66.50	291.47	1007.47	19.382
18	322.49	126.05	66.50	527.77	1475.06	35.096
19	328.47	126.10	70.50	549.85	1515.68	38.765
20	328.46	126.05	66.50	1181.22	2672.09	78.548
21	566.07	126.00	66.50	1693.21	3517.18	112.593
22	351.49	29.20	66.50	1273.32	3121.32	84.673
23	149.55	29.30	34.26	130.59	631.82	4.474
24	358.51	29.20	34.26	1282.58	3137.70	43.941
25	350.36	29.20	116.98	1271.83	3118.68	148.779
26	551.03	29.10	116.98	1552.89	3572.74	181.657
27	301.36	4.50	110.45	1015.40	3068.66	112.149
28	294.54	4.50	124.17	1008.07	3054.61	125.167
29	395.00	35.00	229.58	328.76	805.50	75.475
30	236.65	35.00	229.58	128.61	431.55	29.526
31	237.64	50.00	229.58	130.51	433.68	29.962

**Table 5:** Calculated exergy variables for selected main components.

Component, *k*	${\dot{E}}_{F,k}$ (WM)	${\dot{E}}_{P,k}$ (WM)	${\dot{E}}_{D,k}$ (WM)	$\varepsilon_{k}$ (WM)	$y_{D,k}$ (WM)
Compressor	268.775	250.513	18.262	93.206	2.241
CC	965.762	742.754	223.008	76.909	27.364
Expander	535.666	506.286	29.380	94.515	3.605
Reheater	34.541	32.878	1.663	95.184	0.204
HSH	37.225	34.046	3.179	91.460	0.390
HEV	46.856	43.458	3.398	92.747	0.417
HEC	16.568	15.713	0.854	94.845	0.105
ISH	2.456	2.158	0.298	87.868	0.037
IEV	14.454	13.535	0.918	93.646	0.113
IEC	13.392	12.232	1.160	91.335	0.142
LSH	1.476	1.095	0.381	74.203	0.047
LEV	11.505	10.251	1.255	89.094	0.154
CHP	14.008	9.593	4.415	68.480	0.542
HT	27.920	25.795	2.125	92.388	0.261
IT	69.508	57.739	11.769	83.068	1.444
LT	94.357	82.436	11.921	87.366	1.463
HPFWP	1.015	0.860	0.155	84.757	0.019
IPFWP	0.299	0.247	0.052	82.775	0.006
LPFWP	0.0014	0.0012	0.0002	83.682	0.000
Condensate Pump	0.076	0.060	0.017	78.044	0.002
Condenser	39.933	30.015	9.919	75.162	1.217
De-aerator	17.278	16.686	0.592	96.574	0.073
Solar field	99.720	45.513	54.207	45.641	6.651
Total	814.969	396.685	418.284	48.675	51.325

**Table 6:** Economic analysis of ISCCS.

Investment	Values
Specific investment cost for solar field ($/m^2^)	286.57 [9]
Annual O & M cost (%)	2 [14]
Annual average investment ($/MW)	76.5
Price of natural gas for Industry ($/m^3^)	0.543
Saving fuel cost ($/MW·h)	1.86
Effective discount rate (%)	7 [9]
Economic life (year)	30 [30]
CO_2_ emission reduction (kg/MW·h)	88.40
Net income of system ($/MW)	1097
*LCOE* ($/MW·h)	79.42
Payback time (year)	13.12

## Nomenclature

Abbreviations	
AC	Air Compressor
CC	Combustion Chamber
GT	Gas Turbine
Gen	Generator
ISCCS	Integrated Solar Combined Cycle System
PTSC	Parabolic Trough Solar Collector
CCPP	Combined Cycle Power Plant
HRSG	Heat Recovery Steam Generator
LHV	Lower Heating Value
DNI	Direct Normal Irradiance
HSH	High Pressure Superheater
HEV	High Pressure Evaporator
HEC	High Pressure Economizer
REH	Reheater
ISH	Intermediate Pressure Superheater
IEV	Intermediate Pressure Evaporator
IEC	Intermediate Pressure Economizer
LSH	Low Pressure Superheater
LEV	Low Pressure Evaporator
CPH	Condensate Preheater
HPFWP	High Pressure Feed Water Pump
IPFWP	Intermediate Pressure Feed Water Pump
LPFWP	Low Pressure Feed Water Pump
HT	High Pressure Steam Turbine
IT	Intermediate Pressure Steam Turbine
LT	Low Pressure Steam Turbine
HP	High Pressure
IP	Intermediate Pressure
LP	Low Pressure
$T_{a}$	Ambient temperature
$T_{s}$	Solar surface temperature
$T_{r}$	Collectors surface temperature
$Q_{s}$	Energy received by the collector
$Q_{a}$	Energy absorbed by the absorber
$\dot{E}x$	Exergy of a stream
$\dot{E}x_{ph}$	Physical exergy
$\dot{E}x_{ch}$	Chemical exergy
$\dot{E}x_{i}$	Exergy received by the collector
$\dot{E}x_{c}$	Exergy absorbed by the absorber
$\dot{E}x_{D,k}$	Exergy destruction of *k*th component
$\dot{E}x_{F,k}$	Fuel exergy of *k*th component
$\dot{E}x_{p,k}$	Product exergy of *k*th component
${\dot{E}}_{L,tot}$	Exergy loss in the system
$\dot{E}x_{D,k}^{EN}$	Endogenous exergy destruction of *k*th component
$\dot{E}x_{D,k}^{EX}$	Exogenous exergy destruction of *k*th component
$y_{D,k}$	Exergy destruction rate
Greek Symbol	
$\eta_{e}$	Exergy efficiency rate
$\eta_{\rho }$	Surface reflectivity of collector
$\eta_{\tau }$	Receiver transmissivity of collector
$\eta_{\alpha }$	Receiver absorption of collector
$\eta_{\gamma }$	Acquisition factor of collector
$\eta_{\phi }$	Mirror utilization of collector
$\eta_{\mu }$	Radiation and convective heat loss efficiency of collector
